# Recombinant Interleukin-24 Lacks Apoptosis-Inducing Properties in Melanoma Cells

**DOI:** 10.1371/journal.pone.0001300

**Published:** 2007-12-12

**Authors:** Stephanie Kreis, Demetra Philippidou, Christiane Margue, Catherine Rolvering, Claude Haan, Laure Dumoutier, Jean-Christophe Renauld, Iris Behrmann

**Affiliations:** 1 Laboratoire de Biologie et Physiologie Intégrée (LBPI), Life Science Research Unit, University of Luxembourg, Luxembourg; 2 Ludwig Institute for Cancer Research, University of Louvain, Brussels, Belgium; University Paris Sud, France

## Abstract

IL-24, also known as melanoma differentiation antigen 7 (mda-7), is a member of the IL-10 family of cytokines and is mainly produced by Th_2_ cells as well as by activated monocytes. Binding of IL-24 to either of its two possible heterodimeric receptors IL-20R1/IL-20R2 and IL-22R/IL-20R2 activates STAT3 and/or STAT1 in target tissues such as lung, testis, ovary, keratinocytes and skin. To date, the physiological properties of IL-24 are still not well understood but available data suggest that IL-24 affects epidermal functions by increasing proliferation of dermal cells. In stark contrast to its “normal” and physiological behaviour, IL-24 has been reported to selectively and efficiently kill a vast variety of cancer cells, especially melanoma cells, independent of receptor expression and Jak-STAT signalling. These intriguing properties have led to the development of adenovirally-expressed IL-24, which is currently being evaluated in clinical trials. Using three different methods, we have analysed a large panel of melanoma cell lines with respect to IL-24 and IL-24 receptor expression and found that none of the investigated cell lines expressed sufficient amounts of functional receptor pairs and therefore did not react to IL-24 stimulation with Jak/STAT activation. Results for three cell lines contrasted with previous studies, which reported presence of IL-24 receptors and activation of STAT3 following IL-24 stimulation. Furthermore, evaluating four different sources and modes of IL-24 administration (commercial recombinant IL-24, bacterially expressed GST-IL-24 fusion protein, IL-24 produced from transfected Hek cells, transiently over-expressed IL-24) no induction or increase in cell death was detected when compared to appropriate control treatments. Thus, we conclude that the cytokine IL-24 itself has no cancer-specific apoptosis-inducing properties in melanoma cells.

## Introduction

Interleukin (IL) -24 (mda-7), a member of the IL-10 family of cytokines (together with IL-10, -19, -20, -22, and -26), has been discovered in 1995 by subtraction hybridization following “differentiation therapy” of melanoma cells: through treatment with IFN-β and a protein kinase C inhibitor (mezerein) neoplastic melanoma cells terminally differentiate and lose their proliferative capacity. By comparing gene expression profiles in treated versus untreated cells, mda-7 (amongst others) was identified to be up-regulated in non-proliferative and differentiated melanoma cells [1]. In the following years it was shown that IL-24/mda-7 is basally expressed in tissues related to the immune system (thymus, spleen, macrophages and PBMC's, especially in monocytes and T cells), in normal melanocytes, and keratinocytes [2]–[5]. Genomic localisation, sequence and structural comparisons, as well as expression patterns, led to the renaming of mda-7 to IL-24 [6]. Thereafter it has been demonstrated that IL-24 is in fact a typical cytokine as it binds to specific receptors, it is secreted, it activates the JAK/STAT signaling pathway and it can modulate growth characteristics of responsive cells [7]–[10].

IL-24 (and IL-20) interacts with two different heterodimeric receptor complexes from the class II cytokine receptor family: IL-20R1/IL-20R2 and IL-22R/IL-20R2. On receptor-expressing cells, IL-24 activates the JAK-STAT pathway, shown by phosphorylation of STAT3 [11], [12]. However, the cellular targets of IL-24-activated STAT3 transcription factors and the physiological role of this IL-24-induced signalling remain to be clarified.

A recent and comprehensive study by Kunz and colleagues [2] investigated the expression patterns of IL-24 and its receptor chains by quantitative real-time PCR (qPCR). IL-24 was predominantly expressed by skin tissue cells during local subchronic inflammation but not during acute inflammation. Also, IL-24 expression in skin samples from patients with psoriasis showed no dramatic increase when compared to healthy skin from the same patients. In contrast, IL-19 and IL-20 were strongly induced in samples from patients with inflammatory skin disease while freshly isolated keratinocytes expressed robust levels of IL-24 and IL-20. Interestingly, immune cells did not express receptors for IL-24 suggesting that this cytokine cannot stimulate immune reactions by directly interacting with cells from the immune system [2] but that it rather exerts its functions on keratinocytes, inflamed skin and/or yet unidentified target tissues. Apart from the physiological properties that IL-24 may have under “normal” conditions, far more emphasis has been placed on the tentative ability of this cytokine to selectively induce apoptosis of cancer cells.

The first interest in IL-24 as a possible therapeutic agent for cancer came from observations that IL-24 transcripts or protein were gradually lost during advanced invasive progression of melanoma [13], [14]. Initial evidence on IL-24-induced apoptosis of cancer cells came from Paul Fisher's group who showed that transient transfection of IL-24 into melanoma cells reduced colony numbers while healthy cells remained unaffected by such a paradigm [1], [15]. To improve modes of IL-24 delivery to various normal and cancer cell types, a replication-deficient Adenovirus expressing IL-24/mda-7 (Ad-mda7) was constructed, which, when applied to breast cancer cells, induced selective apoptosis and inhibited breast cancer growth in nude mice [16], [17]. Since the first reports on this apoptosis-inducing function of Ad-mda7 numerous studies have been published demonstrating that IL-24, when administered in supra-physiological amounts via an adenoviral vector, selectively and efficiently kills cancer cell lines derived from breast, cervix, colon, lung, prostate, glioma, melanoma, ovary, bone, and other sources [10], [16], [18]–[25]. These remarkable findings have led to the development of INGN 241 (Introgen), a replication-incompetent IL-24-expressing adenovirus, which is currently in phaseII/III clinical trials (Introgen, company homepage, [26]).

Despite this advanced stage of clinical testing many fundamental questions regarding the tentative IL-24-mediated selective killing of cancer cells remain to be answered. For example, conflicting data exist, concerning the effects of IL-24 expressed and delivered by routes other than the adenoviral over-expression system. IL-24 secreted from transfected human embryonic kidney cells (Hek) was shown to elicit apoptosis of various cancer types *in vitro* [16], [27]–[30] while others reported that this form of IL-24 was unable to alter growth characteristics of cancer cells (Caudell et al., 2002; Pataer et al., 2005; Ramesh et al., 2003). Another source of recombinant IL-24, a GST-fusion protein expressed in and purified from *E.coli* bacteria, has been reported to kill prostate, breast and pancreatic carcinoma cell lines [31], [32], whereas others [28], [33] found no such attributes of GST-IL-24. In this context, it remains completely unclear how these different recombinant forms of IL-24 retain their deduced selectivity for cancer cells while healthy cells are not affected or harmed.

Depending on the type of cancer under investigation many different signalling molecules and pathways are believed to actively partake in the IL-24-mediated killing of target cells (for comprehensive reviews, see [34], [35]). Nevertheless, several studies converge on an UPR (unfolded protein response) or ER (endoplasmic reticulum) stress response, triggered by a temporal over-expression of recombinant IL-24 in the ER, which eventually promotes apoptosis of cancer cells. Surprisingly, the same over-expression regimen does not appear to harm healthy cells [8], [33], [36]. Adding yet another puzzling property to this cytokine it has been reported that infection, transfection or transduction of cells with full length IL-24 leads to secretion of functional cytokine, which then elicits so-called “bystander” effects killing surrounding (receptor-positive) cancer cells [28], [37].

Incited by the tentatively beneficial traits of this IL-10-family cytokine, we initially set out to investigate the signalling events underlying IL-24-induced direct as well as “bystander” killing of melanoma cells. To our surprise, from a panel of 12 different melanoma cell lines, including A375, MeWo, and Wm35, which had previously been described to express receptors for IL-24 and to respond to IL-24 stimulation [28], none reacted to IL-24 stimulation with activation of STAT3. By means of RT-PCR, qPCR, and western blot analysis we show that none of the tested melanoma cell lines expresses a functional IL-24 receptor complex. Finally and importantly, five different routes of IL-24 administration, including intracellular over-expression, were evaluated for their potential to kill a selection of melanoma cell lines. No increased apoptosis was observed in any of the tested scenarios when compared to appropriate control treatments and therefore we conclude that the cytokine IL-24 itself is not sufficient to selectively drive melanoma cells into apoptosis.

## Results

### Lack of Jak/STAT activation in melanoma cells following stimulation with IL-24

**Figure 1:**
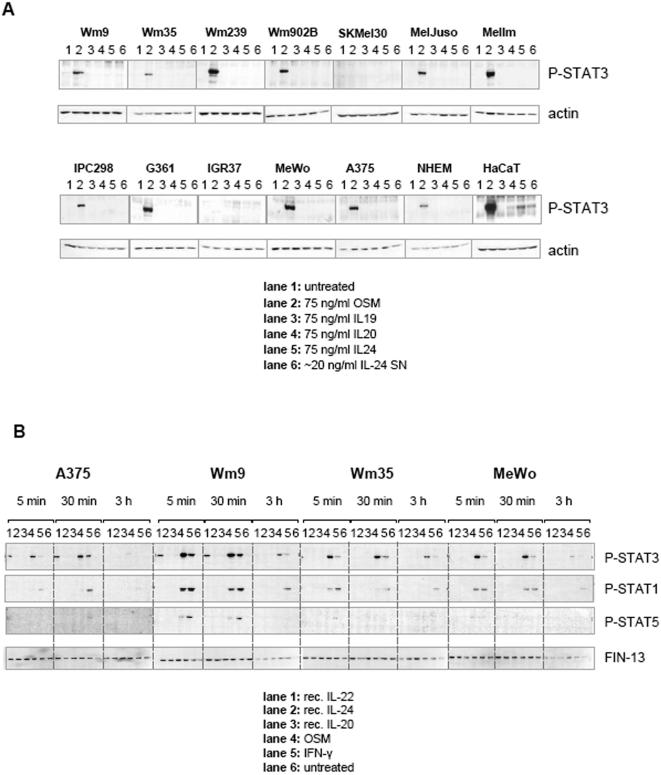
Western blot analysis of melanoma cells stimulated with IL-10-type cytokines IL-19, -20, and -24 shows no induction of STAT phosphorylation. (A) 12 Mycoplasma-free melanoma cell lines, NHEM, and HaCaT cells were grown in 6-well plates and then stimulated for 45 min with the indicated cytokines (75 ng/ml). Cells were lysed directly on ice by addition of 1× Laemmli buffer. Transferred proteins were incubated over night with anti P-Tyr STAT3 antibody, stripped and counter-stained with anti-actin antibody. (B) Stimulation of a selection of melanoma cells was performed as described in (A) only that cytokines were added for different periods of time (5 min, 30 min, 3 h) before cells were harvested. Stripping of western blot membranes prior to incubation with another phospho-specific antibody was performed as described in materials and methods.

Initially, a panel of 12 different melanoma cell lines, the immortalised keratinocyte line HaCaT and primary human melanocytes (NHEM), were evaluated for classic Jak-STAT activation following IL-24 stimulation. In parallel, we analysed related IL-10-type cytokines, IL-19 and IL-20. Fig. 1A shows that none of the 3 recombinant cytokines had any effect on melanoma cell lines, while OSM (lanes 2, positive control) induced a strong phosphorylation of STAT3 in 10 out of 12 cell lines. Interestingly, SKMel30 and IGR37 cells were reproducibly non-responsive to OSM. In agreement with previous reports, HaCaT cells clearly respond to IL-24 (lane 5), to a lesser extent to IL-20 (lane 4) and very weakly to IL-19 (lane 3) [2], [7]. Surprisingly, the melanoma cell lines A375, MeWo, and Wm35 did not react to IL-24 although previous studies had reported that IL-24 led to STAT3 activation in these cells [28], [29]. Additionally, IL-24 produced from transfected Hek cell supernatants (lanes 6), which will be discussed in more detail below, also induced phosphorylation of STAT3 in HaCaT cells but not in any of the melanoma cell lines. Interestingly, primary melanocytes (NHEM, at passage no. 4) did not show an activation of STAT3 following stimulation with the tested IL-10-type cytokines.

To determine if STAT activation, in response to these recombinant cytokines follows a distinct kinetic, we stimulated 4 selected cell lines with 100 ng/ml of IL-24, IL-20, and IL-22 in addition to control treatments with OSM and IFN-γ over three different periods of time (5, 30, and 180 min). Fig. 1B shows that IL-20 and IL-24 had no effect on melanoma lines, while IL-22 induced phosphorylation of STAT3 in A375 and Wm9 cells but not in Wm35 or MeWo. Control treatments with OSM and IFN-γ resulted in the expected activation patterns of STAT3, 1, and 5 [38], [39].

**Figure 2:**
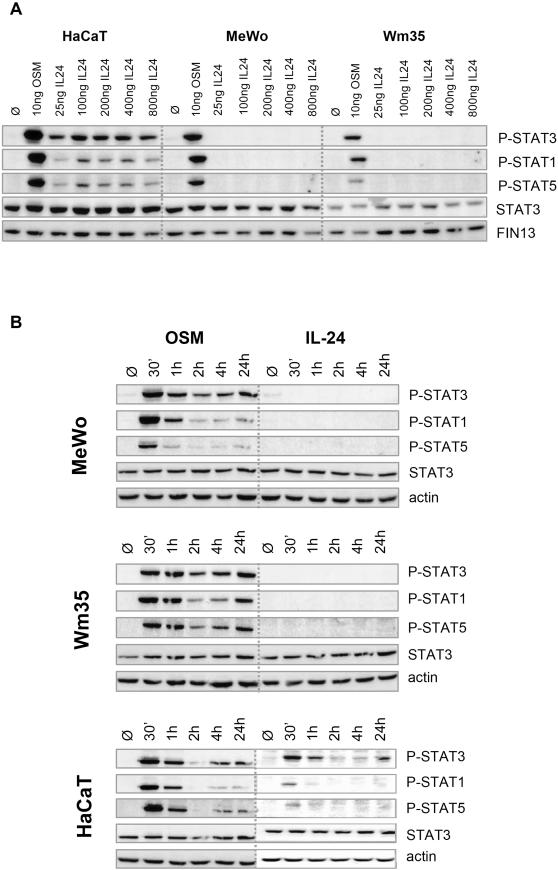
Kinetics and dose-dependence of IL-24 stimulation. HaCaT keratinocytes (positive control cells reacting to IL-24) and 2 selected melanoma cell lines (MeWo and Wm35) were incubated with increasing amounts of IL-24 for 30 min (A) or with 25 ng/ml OSM or 200 ng/ml IL-24 for different periods of time (B). Cell lysates were analysed by western blot with subsequent staining of P-STAT3, P-STAT1, and P-STAT5 followed by STAT3 and actin counter stains.

Focusing on IL-24, we then performed standard dose-response and extended kinetic studies on HaCaT keratinocytes and the two melanoma cell lines MeWo and Wm35. Fig. 2A illustrates that as little as 25 ng/ml IL-24 led to a robust activation of STAT3 and to a much lesser extent of STAT1 and 5 in responsive HaCaT cells. However, concentrations as high as 800 ng/ml did not produce any STAT phosphorylation in the two assayed melanoma cell lines. Even upon heavy over-exposure of some of the western blots did we not see potentially weak IL-24-induced P-STAT3 signals (data not shown). The possibility of a delayed STAT activation following IL-24 treatment was ruled out by incubating the cells for up to 24 h (Fig. 2B). Control treatments with OSM showed a typical STAT phosphorylation pattern, which decreases at around 2h, the time when SOCS proteins (suppressor of cytokine signalling) become active [40].

### Expression of IL-24 and its receptors on melanoma cells

Classic cytokine signalling events require the binding of a cytokine to its receptor(s), an event which results in the transphosphorylation and activation of Jaks. Subsequently, active Jaks phosphorylate tyrosine residues within the receptor cytoplasmic domain, which form docking sites for STATs. Jaks then tyrosine-phosphorylate receptor-bound STATs so that active STAT dimers can finally translocate to the nucleus where they initiate transcription by binding to response elements in the promoter of a target gene [40].

**Figure 3:**
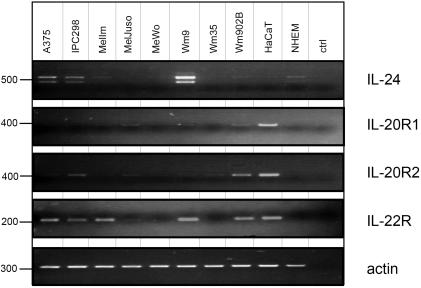
RT-PCR of IL-24 and its receptor subunits. Eight different melanoma cell lines, primary NHEM and HaCaT cells were grown on Petri dishes to a confluence of 80–90% before cells were harvested. Total RNA was extracted, reverse transcribed and cDNA amounts equivalent to 100 ng RNA were used as input material for amplifications of IL-24, and the receptor subunits IL-20R1, -20R2, and -22R. To control for equal starting amounts and loading, actin was amplified. Size markers (bp) are indicated on the left.

**Figure 4:**
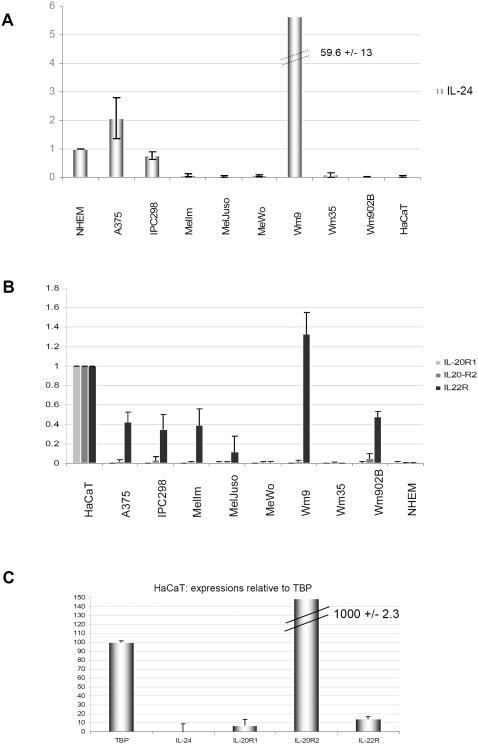
Quantitative PCR for IL-24 and its 3 possible receptor subunits: absence of a functional IL-24 receptor on melanoma cells. RNA was extracted and reverse transcribed as for standard RT-PCR. 12.5 ng RNA was used as input material followed by specific amplification of target genes as indicated. Results are means +/− STD of triplicate amplifications from three independent experiments using 3 independently extracted RNAs. Ct values were normalized to those of the housekeeping gene TBP for each tested cell line. Calculations of relative expression values were performed using the 2^−ΔΔCt^ formula and expression levels were correlated to NHEM results for IL-24, (A) and to HaCaT for receptor subunits, (B), which were set to 1. (C) The Ct value of the housekeeping gene TBP (in HaCaT cells) was arbitrarily set to represent 100 mRNA copies. The Ct values of IL-24 and the three receptor subunits were converted accordingly in order to facilitate a clearer view of the relative amounts of the 4 target genes compared to the housekeeping gene TBP.

IL-24 signals through two heterodimeric receptor complexes, which share the short IL-20R2 chain but have distinct longer alpha chains: IL-20R1 and IL-22R [7]. Since no Jak/STAT signalling was detected in any of the melanoma cell lines following IL-24 (or IL-19 and IL-20) treatment, the expression of the 3 possible receptor chains was analysed by standard RT-PCR, quantitative PCR (qPCR), Western Blot and FACS analyses. Primers used for PCR reactions are listed in Table 1. First, standard RT-PCR was performed using the equivalent of 100 ng RNA as starting material (Fig. 3). Some but not all cell lines showed positive results for IL-22R mRNA while hardly any mRNA for IL-20R1 and very low or absent mRNAs for IL-20R2 chains, the common subunit present in both heterodimeric receptor complexes, were detected. As expected and as previously published, HaCaT cells were positive for all 3 receptor chains but negative for IL-24. IL-24 mRNA was detectable to various levels in 6 out of 10 melanoma cell lines. The lower band present in IL-24 RT-PCR samples corresponds to a splice variant, which has been described before [41]. Previous reports have suggested that melanoma cells express receptors for IL-24 although it is unclear how this was analysed [28]. Performing RT-PCR with very high cDNA input quantities (equivalent to 1 µg of RNA), we also found all analysed cell lines to be positive for the 3 possible IL-24 receptor chains (data not shown). However, lower starting amounts of cDNA (10-100 ng/ml) reproducibly gave results as depicted (Fig. 3) and these results matched well with those from qPCR shown in Fig. 4. Again, in depth qPCR analysis was performed on different amounts of input material to achieve a standard curve and a PCR amplification efficiency of 100% for all samples. Fig. 4 shows a summary of triplicate qPCR data from 3 independent experiments using the equivalent of 12.5 ng RNA input material. mRNA amounts of IL-24 were normalised to TBP and are shown in correlation to NHEM (Fig. 4A). A375 and IPC298 showed moderate amounts of IL-24 mRNA with Wm9 reproducibly having IL-24 mRNA quantities ∼50 fold higher than primary melanocytes.

**Table 1:** Summary of primers used for quantitative real time PCR, standard RT-PCR amplifications and for IL-24 cloning.

Target gene	sense primer	antisense primer	application	size (bp)
**IL-20R1**	TGTTGCTCCTGGCGG	TTGATGGATAAGAAGGTGATGTTTG	qPCR	91
	TCAAACAGAACGTGGTCCCAGTG	TCCGAGATATTGAGGGTGATAAAG	RT-PCR	386
**IL-20R2**	TGTTGCCCCGTGGTGGT	CTCCTCCCTTCTGCAGCTGAT	qPCR	81
	GCTGGTGCTCACTCACTGAAGGT	TCTGTCTGGCTGAAGGCGCTGTA	RT-PCR	406
**IL-22R**	GAAGTCCTGCAACCTGACG	GGTAGTGTGCTGCAGAGAGC	qPCR	133
	CCCCACTGGGACACTTTCTA	TGGCCCTTTAGGTACTGTGG	RT-PCR	243
**IL-24**	CAGGAGGAACACGAGACTGA	GCACAACCATCTGCATTTGAGA	qPCR	120
	CAGTCGACACCATGAATTTTCAACAGAGGCTGC	GAGGATCCCAGAGCTTGTAGAATTTCTGCATC	RT-PCR	639
**TBP**	ACCCAGCAGCATCACTGTT	CGCTGGAACTCGTCTCACTA	qPCR	120
**Actin**	GCTCGTCGTCGACAACGGCT	CAAACATGATCTGGGTCATCTTCTC	RT-PCR	353
**IL-24**	CGGATCCGTACCATGAATTTTCAACAG	CTGCTCGAGTCAGAGCTTGTAGAATT	cloning of GST-mda7	
**IL-24**	GCTCCTGCAGGTACCATGAATTTTCAACAG	CTCCACCGGTGAGAGCTTGTAGAATTTCTGCATCC	cloning of KDEL-IL24	

Note: primers for amplification of IL20R1, R2, and IL-24 were as described by Wolk et al., 2002.

Recently, it has been shown that primary keratinocytes harbour high mRNA amounts for IL-20R2 and approximately 10-fold less for IL-22R followed by very low levels of IL-20R1 mRNA [2]. Here, we found very similar expression patterns for the keratinocyte cell line HaCaT. To better exemplify these largely divergent levels of the receptor chain mRNAs, copy numbers relative to those of the house-keeping gene TBP, arbitrarily set to 100, are depicted in Fig. 4C. This representation illustrates that HaCaT cells have approximately 10× more IL-20R2 than TBP mRNA while IL-22R and IL-20R1 would correspond to mRNA copy numbers of ∼15 and ∼10, respectively, relative to the arbitrarily chosen 100 copies for TBP. Following this theoretical exercise, it becomes evident that the melanoma cell lines analysed here do not have significant amounts of IL-24 receptor subunit mRNAs.

**Figure 5:**
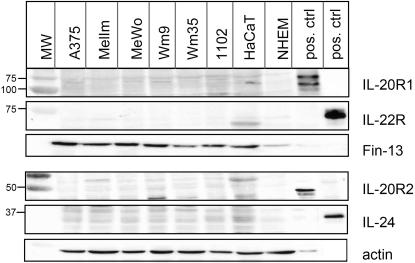
Western blot analysis of IL-24 and its receptors in total melanoma cell lysates. Total cell lysates of the depicted melanoma cell lines, NHEM and HaCaT cells as well as control samples were analysed by Western blot to detect endogenous levels of IL-24 and its receptors. Positive control samples: 100 ng of commercial recombinant IL-24 or Hek cells transiently transfected with the respective receptor subunit plasmids pcDEF3-IL-20R1, pCEP4-IL20R2, or pEF-BosPuro-IL-22R as described before[7]. Lysates were loaded in parallel on either an 8% gel (IL-20R1 and IL22R) or a 10% gel for detection of IL-20R2 and IL-24. After the first round of detection, blots were stripped and re-probed over night at 4°C with subsequent antibodies. As transfected cells were strongly over-expressing the individual receptor chains, less lysates were loaded for the controls (see loading controls stained with actin or FIN13). Commercially available antibodies recognize all positive controls but fail to detect specific bands correlating to endogenous levels of receptors or IL-24.

Since levels of mRNA do not necessarily reflect actual protein expression, we analysed total cell lysates for the presence of IL-24 and its receptors by western blot (Fig. 5). Commercially available antibodies recognised positive controls (Hek cells over-expressing the respective receptor subunits and recombinant IL-24, R&D Systems) at the expected molecular weights. However, endogenous levels of surface receptors, even in HaCaT cells, were too low to be detectable by western blot. For better comparison, lysates from transfected, over-expressing cells had to be diluted 5 to 10-fold (Fig. 5: see loading controls stained with FIN-13 or actin antibodies). We also performed FACS analysis but could not detect any of the three IL-24 receptor subunits on melanoma cells, HaCaT or NHEM cells using commercially available antibodies (data not shown).

Taken together, melanoma cell lines evaluated in this study did not express sufficient amounts of specific receptor pairs, which could mediate effects brought about by the cytokine IL-24. Hence, the “bystander” effect that secreted IL-24 has been reported to have on receptor-positive cancer and melanoma cells [34], [42] is difficult to reconcile with the lack of receptors found here.

### Recombinant IL-24 has no effect on the proliferation of melanoma cells

**Figure 6:**
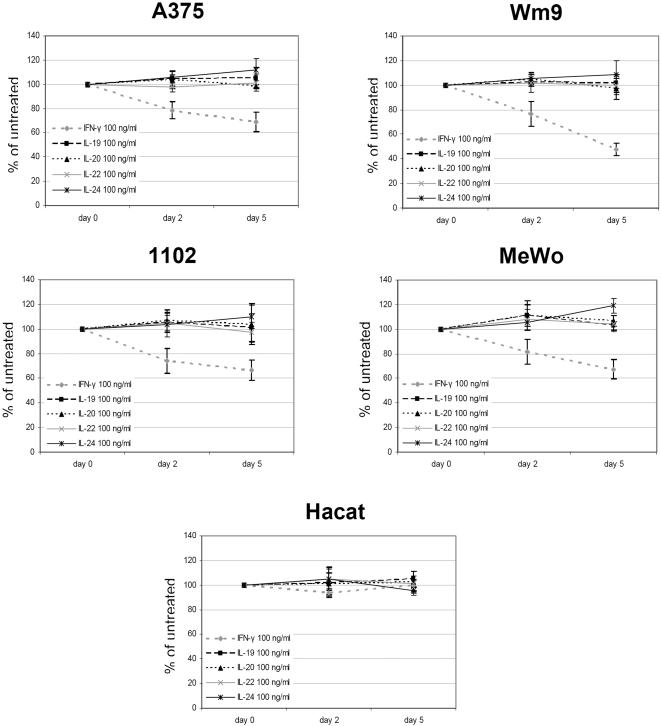
Influence of IL-10-type cytokines on the proliferation of melanoma cells. Four different melanoma cell lines and HaCaT cells were seeded in their appropriate growth media supplemented with 1% FCS (96-well plates) and were incubated with 100 ng/ml of IL-19, -20, -22, -24 or IFN-γ. At day 0, 2, and 5, 5 µl of WST-1 reagent was added for 40 min to each well before absorbance at 450 nm was measured. Shown are results of triplicate wells from three different experiments +/− STD.

Exogenously administered recombinant cytokines (IL-19, -20, -22, -24, and IFN-γ) were analysed with regard to their growth-modulating properties on a selection of melanoma cells (Fig. 6). No growth-inhibiting or -stimulating effect was monitored when cells were followed up for 5 days. IFN-γ, which was included as a control treatment known to inhibit the proliferation of melanoma cells [39], showed the expected effects. Although functional receptors are present on HaCaT cells, none of the assayed cytokines influenced proliferation of the keratinocyte cell line. HaCaT cells were confirmed by qPCR to express moderate levels of IL-10R2, a receptor chain, which, together with IL-22R, is required for IL-22-signalling (data not shown).

Having established that commercially available recombinant cytokines expressed in mouse myeloma NSO cells (IL-24) or bacteria (IL-19, -20) have no growth-modulating effect on melanoma cells, we continued to examine different sources of recombinant IL-24 for their impact on melanoma cells.

**Figure 7:**
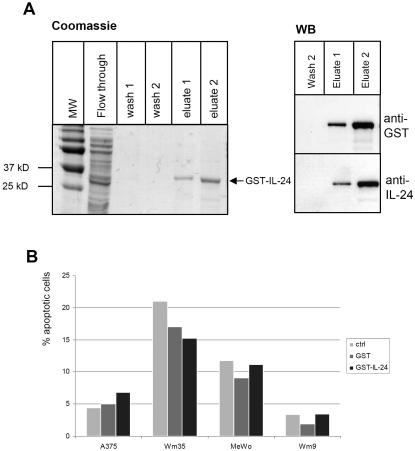
Recombinant GST-IL-24 and its effect on the growth of selected melanoma cells. (A) GST-IL-24 was cloned and purified as described in materials and methods. Samples from the flow through, wash and eluted fractions were analysed on a SDS-PAGE followed by Coomassie staining. Western blots of recombinant GST-IL-24 showing specific staining with anti-GST and anti-IL-24 antibodies. (B) AnnexinV-apoptosis assay on cells that were incubated with 40 µg/ml recombinant fusion proteins for 48 h. GST-IL-24 does not substantially increase the number of apoptotic melanoma cells relative to control treatments.

Sauane and colleagues [31] have shown that a GST-IL-24 fusion protein was able to induce apoptosis of prostate, pancreas and breast cancer cell lines. Following this report, we produced full-length GST-IL-24 to examine the fusion proteins' potential to drive melanoma cells into apoptosis. After considerable modifications to the published purification protocol in order to achieve soluble protein, we were able to produce small quantities of pure GST-fusion protein (Fig. 7A). Up to 250 ng/ml of this fusion protein did not evoke a STAT3 phosphorylation in otherwise IL-24-responsive HaCaT cells (data not shown), which was not surprising given the presence of a bulky GST tag and the likely altered conformation of the cytokine in such a recombinant fusion protein. We then tested the effects of GST-IL-24 in comparison to GST alone and untreated cells in Annexin V apoptosis assays. Fig. 7B illustrates representative results on A375, Wm9, Wm35 and MeWo cells (results were reproduced 2× with different batches of GST-IL-24). No substantial and reproducible induction of apoptosis was detected after 48 or 72h of treatment in any of the assayed melanoma cell lines. Hence, in our hands, bacterially produced GST-IL-24 fusion protein did not induce notable apoptosis of melanoma cells.

### Assessment of IL-24-apoptosis-inducing properties by transient over-expression

**Figure 8:**
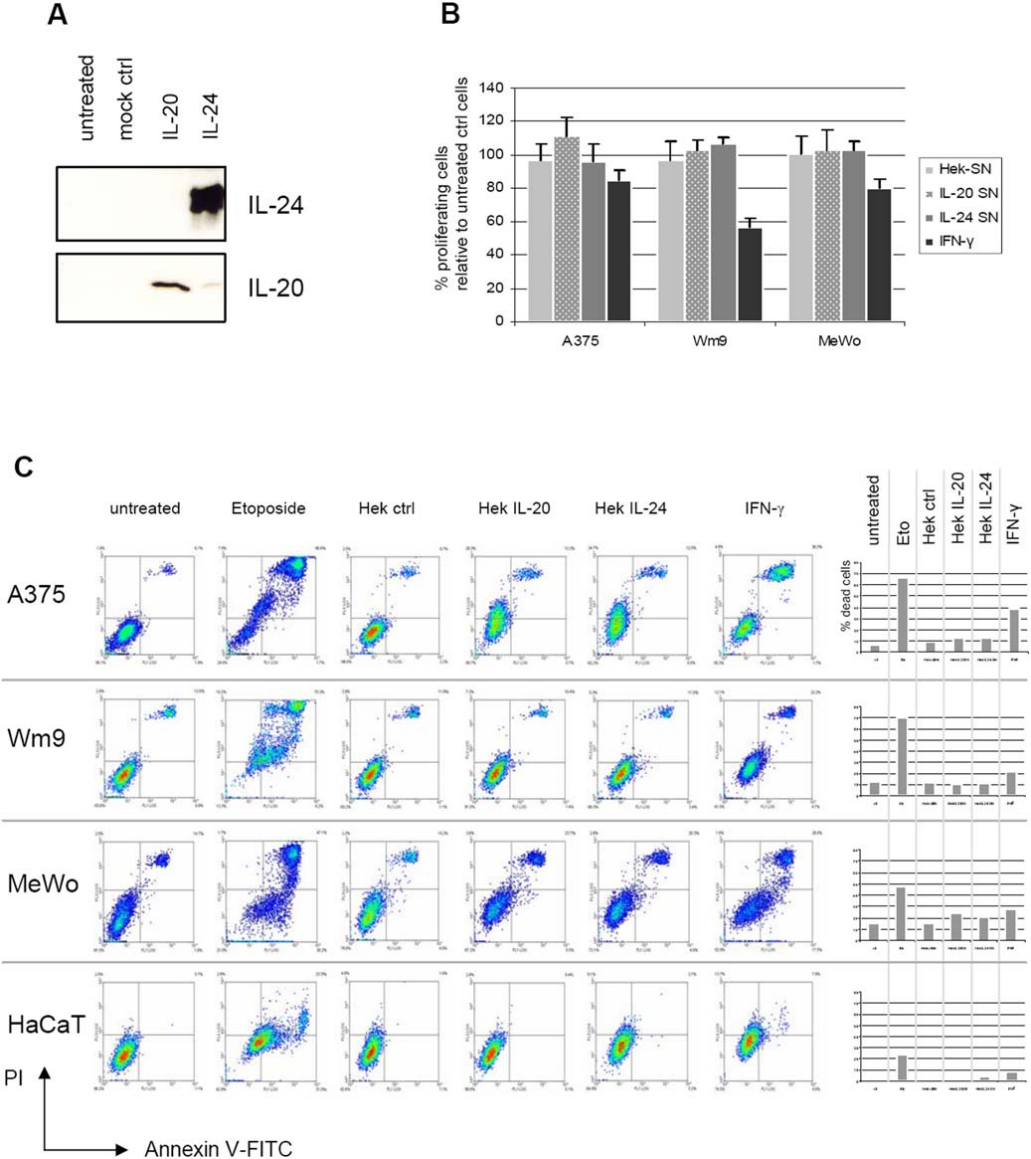
Mammalian IL-24 secreted and purified from transfected Hek cells does not induce apoptosis in melanoma cells. (A) Western blot analysis of supernatants from Hek cells transfected with mock vector pCEP, pCEP-IL-20, or pCEP-IL-24. (B) Proliferation assay of 3 selected cell lines that were incubated for 72 h with at approximately 40 ng/ml of IL-20 or IL-24 from transfected Hek cell supernatants. (C) Density blots of FACS apoptosis assay of selected cell lines incubated for 72 h with the indicated Hek supernatants, IFN-γ, or Etoposide, which served as positive control inducing profound apoptosis in most cell lines. The percentage of dead cells is depicted as bar diagrams on the right panel. Neither Hek-produced IL-20 nor IL-24 increase apoptosis in melanoma cells.

Since we did not detect any apoptosis-inducing properties of either the bacterially produced GST-IL-24 or the commercially available IL-24 (from mouse cells), we proceeded to produce FLAG-tagged IL-24 from supernatants of transfected Hek cells (Hek-IL-24) as has been described before (Fig. 8A) [7]. This mammalian source of recombinant and soluble IL-24 has previously been reported to elicit direct apoptosis as well as bystander effects in melanoma and other cancer cells [30], [34] while other studies failed to detect these effects [33], [43].

In agreement with the latter, we did not observe increased apoptosis or inhibition of proliferation in A375, Wm9, MeWo or HaCaT cells following incubation with crude Hek supernatant containing approximately 40 ng/µl of recombinant IL-24 (Fig. 8B and C) or with further affinity-purified FLAG-IL-24. In all experiments, the growth inhibiting properties of Etoposide and IFN-γ were far more pronounced than any source of IL-24. As a specificity control, we used IL-20, a related IL-10-type cytokine. Neither one of the two soluble and extracellularly administered cytokines had growth-modulating properties on melanoma cells. Additionally, we incubated Wm35 cells with Hek-IL-24 supernatants for 72 or 96h and counted dead cells after Trypan Blue exclusion. No increase in apoptosis was observed (data not shown).

**Figure 9:**
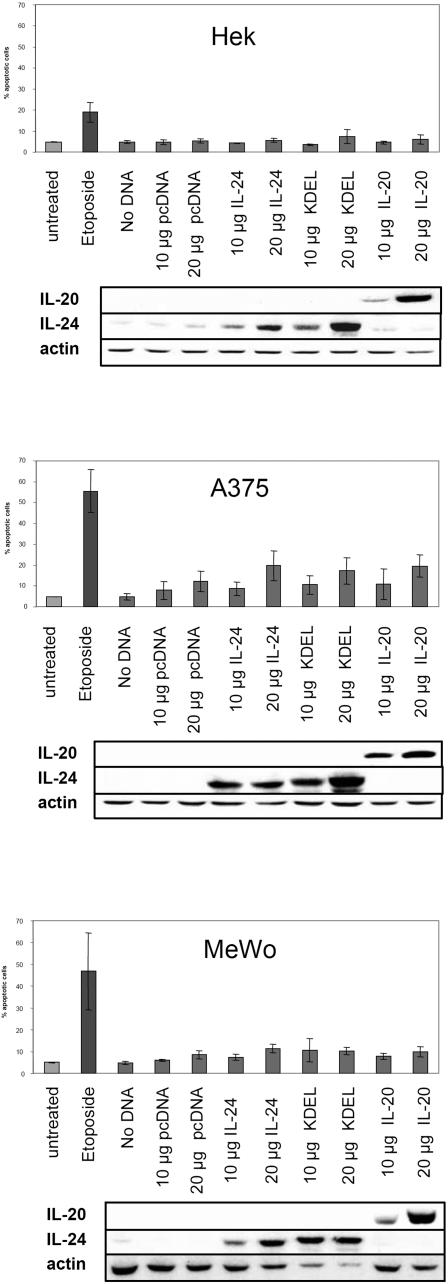
Transient over-expression of IL-24 and ER-targeted IL-24 does not increase apoptosis. Cells were transiently transfected with 2 different amounts of DNA encoding mock vector alone (pcDNA), pcDNA-IL-24 (IL-24), pcDNA-KDEL-IL-24 (KDEL) or pcDNA-IL-20 (IL-20) using nucleofection technology from Amaxa 720h after transfection, cells were processed for AnnexinV-staining detected by FACS. Percent dead cells are depicted in the bar diagrams (duplicates from 3 independent experiments). Below, control western blots show the specific over-expression of the respective proteins.

Finally and to test the possibility that only intracellularly over-expressed IL-24 would render cancer cells more susceptible to apoptosis, we transiently transfected melanoma and Hek cells with either full length IL-24 or IL-20 as well as with a construct that contains full length IL-24 together with a KDEL ER-targeting sequence (KDEL) as several studies have recently postulated that the apoptosis-inducing effects of IL-24 are mainly mediated by triggering ER stress responses [33], [36]. This has been shown extensively using IL-24 that was delivered to cells by an adenoviral expression system. Depending on the multiplicity of infection, it can be speculated that IL-24 is largely over-expressed from within the cell by such a regimen and this is likely to induce ER stress responses, which culminate in cell death. Using transient over-expression but explicitly circumventing the adenoviral backbone, we have used standard pcDNA5 vectors to introduce the recombinant proteins into cells. Fig. 9 depicts results for 2 melanoma cell lines and Hek cells, which served as a negative control as these non-cancer cells should not die under the influence of IL-24 [36]. Etoposide, a potent inducer of apoptosis, was included as a positive control for the Annexin V-FACS assays. Although there is a dose-dependent increase of cell death, especially in A375 cells, this increase was also observed for control transfections (IL-20 and pcDNA alone). Western blots below the bar diagrams illustrate that recombinant proteins were dose-dependently over-expressed in the respective target cell lysates. Overall and relative to control treatments, no cancer-specific apoptosis-inducing properties of IL-24, even when over-expressed from within melanoma cells, were recorded.

## Discussion

Over the past decade numerous publications have argued that IL-24, an IL-10-family cytokine, selectively kills a vast variety of cancer cells, *in vivo* and *in vitro*, leaving healthy cells unharmed (for recent reviews: [8], [34]). These astonishing attributes have served as a basis for the development of a therapeutic adenovirus delivering IL-24 (Ad-IL-24) to cancer patients, a form of treatment that is currently being assessed in clinical trials (Introgen company homepage). It is believed that the combination of IL-24 with adenoviral over-expression represents a promising treatment option for various solid cancers [44]. However, conflicting data exist as to whether other recombinant forms of IL-24 (bacterial GST-fusion protein, baculovirus-expressed IL-24, secreted IL-24 from transfected mammalian cells) have this apoptosis-inducing property and whether these effects are receptor-dependent or not. By using cell lines deficient of individual components of the Jak/STAT pathway or by using Jak inhibitors it was demonstrated that initial IL-24-induced apoptosis of cancer cells is independent of Jak/STAT signaling pathways and thus independent of receptor engagement [10], [45]. However, there is also evidence that IL-24 mediates bystander effects via receptor-mediated Jak/STAT signaling on receptor-positive surrounding cancer cells that come into contact with the secreted cytokine IL-24 [10], [27], [28].

Focusing on melanoma cells and on the “cytokine-like” properties of IL-24, we first stimulated a panel of 14 different cell lines with commercially available recombinant IL-24. Surprisingly, none of the tested cell lines, except HaCaT keratinocytes, showed phosphorylation of STAT3, also not 3 cell lines (A375, Wm35, and MeWo) that had previously been described to be receptor-positive and to react to an IL-24 stimulus with STAT3 activation. Activation of STAT3 had, however, been scored by a rather unspecific immunofluorescence assay, in which translocation of STAT3 to the nucleus was enumerated [28]. Here we show that melanoma cells did not respond to IL-24, neither when up to 800 ng/ml of cytokine was administered, nor over different periods of time. This led us to investigate receptor expression on these cells. Using standard RT-PCR, qPCR, Western Blot and FACS analysis, we could not detect mRNA or protein quantities sufficient to qualify for a functional IL-24 receptor. Consequently, our cytokine stimulation data correspond to the receptor expression studies: HaCaT keratinocytes express (to various degrees) IL-20R1, IL-20R2, and IL-22R receptor chains and readily react to IL-19, IL-20, and IL-24 stimulation with phosphorylation of (predominantly) STAT3. In contrast, none of the melanoma cell lines expressed sufficient amounts of receptors for IL-10-type cytokines (IL-19, -20, and -24) and do therefore not react to these cytokines by activation of the Jak/STAT pathway.

In agreement with Kunz et al. [2], we also found that keratinocytes express the IL-20R2 chain at very high levels (10 fold higher than the reference gene TBP). Much lower (0.1 fold TBP) was the expression of IL-22R followed by even lower amounts of IL-20R1 suggesting that STAT3 activation of cultured keratinocytes is mediated by engagement of the type II receptor IL-22R/IL-20R2. IL-19 only signals through the type I receptor IL-20R1/IL-20R2 and since IL-20R1 is the limiting subunit on HaCaT cells, IL-19 evoked the weakest P-STAT3 signals. However, and in contrast to a recent report [2], we also observed a weak but detectable activation of STAT1 and STAT5 following 30 min of IL-24 or IL-20 stimulation of HaCaT cells. This could be due to the higher amounts of IL-24 that were used in our assays (100 ng/ml versus 10 ng/ml) (Fig. 2).

The receptor chains IL-20R1 and IL-22R seem to be widely expressed in many tissues, nevertheless, expression of a functional receptor for IL-24 depends on the presence of IL-20R2, the common chain for both receptor pairs [7], [46]. Expression of IL-20R1 has been reported in tissues such as skin, lung, testis, and ovary [45], however, most of the IL-24 receptor expression studies relied on RT-PCR, which is neither quantitative nor does it establish the presence of corresponding proteins and as such, functional receptors. Relative to primary NHEM cells, only 3 melanoma cell lines were found to express IL-24 (A375, IPC298, and Wm9). Interestingly, Wm9, a cell line established from a late stage metastatic melanoma patient [47], harbours very large mRNA quantities for IL-24 while Wm35, an early stage cell line derived from the same patient hardly showed any expression of IL-24, indicating that IL-24 expression is increased rather than decreased during melanoma progression. This contrasts with previous reports where IL-24 was shown to disappear with progression of melanoma in primary patient samples [13], [14]. However, Wm9 and Wm35 were the only paired samples available for the current study and expression patterns of IL-24 and other proteins might be different between established cell lines and primary material.

Considering the calculations given by Kunz et al. [2] together with our very similar qPCR results it is sensible to assume that melanoma cells (and also immune cells) do not express functional receptor pairs that could transmit signals emanating from IL-24, a notion which is further substantiated by the lack of STAT3 activation in these cell types following IL-24 treatment. This throws into doubt whether IL-24 can exert so-called bystander effects on melanoma cells as these are believed to depend on the presence of receptors [28], [30].

To establish whether the cytokine IL-24 itself has the ability to induce apoptosis of cancer cells, we reasoned that delivery modes other than adenovirus-mediated over-expression of IL-24 should also be able to elicit cell death. Sauane and colleagues [32] have argued that GST-IL-24 is unspecifically taken up by target cells via the GST moiety [48] and that this recombinant fusion protein specifically kills cancer cells of different origins. To investigate this further, we produced bacterially expressed GST-IL-24, which became soluble only with major modifications to the published protocol [31] (and Materials and Methods in this paper). GST-mda7 failed to significantly modulate growth characteristics of 4 different melanoma cell lines (Fig. 7).

Next, we tested the properties of IL-24 produced from transfected Hek cells. Using crude or affinity purified Hek-IL-24, again no increase in apoptosis or reduced proliferation was observed relative to untreated cells or cells treated with accordingly produced IL-20 (Fig. 8) and this was not surprising as the tested melanoma cell lines did not express functional IL-24 receptors (Figs. 3 and 4). The Hek-IL-24 was, however, able to evoke phosphorylation of STAT3 in receptor-positive HaCaT cells (Fig. 1A) indicating the functionality of this form of cytokine.

Transient or stable inducible over-expression of proteins can trigger ER stress responses, which ultimately drive the host cell into apoptosis [49]. Depending on the multiplicity of infection (moi), the efficient delivery of IL-24 by an adenoviral expression system is likely to induce UPR or ER stress and several reports have already confirmed this notion. In view of this, it has been shown that Ad-IL-24 directly interacts with the chaperone BiP/Grp78, a major player in ER stress response pathways [36]. Also, transduction of lung cancer cells with Ad-IL-24 increased expression of ER stress-related proteins such as BiP/Grp78, GADD34, XBP-1 and transfection of cancer cells with ER-targeted IL-24 plasmids elicited increased apoptosis compared to proteins targeted to the nucleus or membrane [33]. Transient intracellular over-expression of IL-24 by methods other than viral delivery should therefore result in increased cancer cell death provided that IL-24 itself has the ability to specifically initiate events necessary for apoptosis. Since transfection efficiencies of melanoma cells are generally very low, we first tested several commonly used reagents and protocols. Quantification of transfected cells was performed following transient transfection of recombinant GFP or GFP-IL24 and subsequent FACS analysis or visual counting of fluorescent cells under the microscope. Nucleofection in combination with optimised cell line-specific protocols (Amaxa) yielded highest efficiencies (20–30%) with very little spontaneous cell death (data not shown). Although these transfection efficiencies were acceptable for melanoma cells, they are, however, not optimal and certainly far lower than those achieved with adenoviral expression of IL-24. Nevertheless, 20–30% transfected cells should be sufficient to detect specific and significant increases of apoptosis. Using nucleofection, we introduced wt-IL-24, ER-targeted IL-24 (KDEL) as well as wt-IL-20 into A375 and MeWo cells. Hek cells served as a non cancer control cell line that should be resistant to IL-24-mediated killing. Even up to 20 µg of transfected IL-24 DNA did not result in a surplus of apoptotic cells compared to control experiments, once more demonstrating that even intracellular over-expression does not lead to a specific killing of melanoma cells in response to full length or ER-targeted IL-24.

In summary, the ability of IL-24 to selectively and efficiently induce apoptosis of melanoma and many other cancer cells could not be reproduced using either commercial IL-24, bacterially expressed GST-IL-24, IL-24 secreted from transfected Hek cells, or transient over-expression of different IL-24 constructs. As none of the assayed melanoma cell lines expressed functional receptor pairs for IL-24, it was not surprising that Hek-IL-24 did not trigger increased cell death and in view of this, it seems questionable whether secreted IL-24 from transfected or infected cells could induce so-called bystander effects on receptor-negative melanoma cells. Finally, we could not detect significant cell death upon intracellular over-expression of IL-24 suggesting that the beneficial outcome seen after transduction of cancer cells with Ad-IL-24 may be due to combinatorial effects of IL-24 over-expression achieved via cancer-targeted adenovirus infection only. Adenoviral infection may lead to an upregulation of interferons and/or IL-24 receptors, which, in synergy with over-expressed IL-24 could possibly account for the previously observed therapeutic effects of Ad-IL-24. In view of this, care should be taken when attributing the cytokine IL-24 itself with apoptosis-inducing properties on melanoma cells.

## Materials and Methods

### Cell culture

In total, 12 mycoplasma-free melanoma cell lines were analysed: A375 (ATCC, Wesel, Germany), Wm9, Wm35, Wm239, Wm902B, SKMel30, MelJuso, MelIm, IPC298, G361, IGR37, and MeWo. All cell lines were cultured in RPMI, supplemented with 10% FCS, 50 µg/ml penicillin, 100 µg/ml streptomycin, 0.5 mM L-glutamin in a humidified atmosphere with 5% CO_2_. The keratinocyte cell line HaCaT was grown in DMEM supplemented with 10% FCS, 50 µg/ml penicillin, and 100 µg/ml streptomycin; normal human melanocytes (NHEM) (Clonetics, Cambrex Bio Science, Belgium) were maintained according to the supplier's instructions in complete MGM-4 medium. Hek293 cells were grown in DMEM (supplemented with 10% FCS and antibiotics as above). All cultured cells were routinely tested to be negative for mycoplasma contamination.

### Reagents and antibodies

The following antibodies were used for protein detection in western blots: STAT3, STAT1 (BD Transduction Laboratories, BD Biosciences, Erembodegem, Belgium); STAT5 (Santa Cruz); P-STAT3-Tyr705; P-STAT1-Tyr701, P-STAT5-Tyr694 (Cell Signaling Technologies, Frankfurt, Germany). HRP-labeled secondary antibodies were purchased from Dako (Heverlee, Belgium). Antibodies directed against Fin13 (BD Transduction Laboratories, BD Biosciences) or actin (Chemicon) were used as loading controls where applicable.

For stimulation experiments, cells were treated for the indicated times with different concentrations of recombinant cytokines IL-19, -20, -22 (R&D Systems, produced in E. coli), IL-24 (R&D Systems, produced from mouse myeloma cells), OSM, IFN-γ, IFN-α (Peprotech, London, UK), GST-IL-24 or Hek-IL-24 (see below).

### Western Blot

Cells were seeded in appropriate culture vessels and were grown to approximately 90% density, washed 1× with PBS, followed by direct lysis on ice with Laemmli buffer (20% glycerine, 10% ß-mercaptoethanol, 4% SDS, 0,125M Tris-HCl, pH 6.8, 0.002% bromophenol blue); generally 400 µl were used for 1 well from a 6-well plate. Alternatively, cells were lysed in lysis buffer (1% Triton X-100, 20 mM Tris-HCl, pH 7.5, 150 mM NaCl, 10 mM NaF, 1 mM Na_3_VO_4_, 10 mM PMSF, 1 mM benzaminidine, 5 µg/ml aprotinin, 3 µg/ml pepstatin, 5 µg/ml leupetpin) and the concentration of total cell lysates was determined by Bradford assay (BioRad, California, USA). Fifty µg of total lysates were boiled for 5 min at 95°C and loaded on a 10% SDS-PAGE gel. Proteins were transferred onto a PVDF membrane and detected with the relevant antibodies. Before re-probing, membranes were stripped for 30 min at 70°C in 62.5 mM Tris, 2% SDS, pH 6.7 and 78 µl ß-mercaptoethanol/10 ml buffer. Alternatively, for re-probing with phospho-specific antibodies, blots were stripped for 4–5 h at room temperature in 2 M glycine, pH 2.5 with 1 g of SDS for each blot, washed extensively in H_2_O, blocked for 1 h (TBS+5% BSA), and incubated with the next antibody. For highly sensitive detection of chemiluminescent signals, the ECL solution pCA was used as described [50].

### Proliferation and apoptosis assays

For proliferation assays 3000 cells/well were seeded in quadruplicate wells (96 well plates). Increasing concentrations of cytokines or other compounds were added for different lengths of time as indicated. WST-1 reagent (5 µl/well) (Roche Biochemicals, Basel, Switzerland) was added for 30 min at 37°C and absorbance was measured at 450 nm.

For apoptosis assays, 750 000 cells were grown in quadruplicate in 6-well plates with addition of GST, GST-IL-24, Hek-control supernatant (Hek-ctrl), Hek-IL-24 or commercial recombinant cytokines for 48h or 72h. Cells were harvested and processed as described in the Annexin V-FITC Apoptosis Detection kit II manual (BD Transduction Laboratories, BD Biosciences, Erembodegem, Belgium). FITC and Propidiumiodide fluorescences were measured using a BD FACS Canto II (Becton Dickinson). Alternatively, cells were transiently transfected with pcDNA vector (mock control), pcDNA expressing full length IL-24, full length IL-20, or a pcDNA construct expressing IL-24 together with a KDEL sequence, which targets recombinant proteins to the endoplasmic reticulum (ER). Cells were transfected using the Nucleofector technology (Amaxa, Cologne, Germany) according to optimised protocols provided by Amaxa for Hek, A375 and Mewo cell lines and apoptosis was measured as described above after 48 and 72h. To control for successful transient expression of transfected proteins, correspondingly treated samples were washed with PBS and lysed directly on ice with 1× Laemmli buffer followed by western blot analysis.

### Gene expression analysis

Total RNA was extracted from cells grown on a 10 cm petri dish (∼90% confluent) using the RNeasy Mini Kit (Qiagen, Hilden, Germany) according to the manufacturer's instructions with additional on-column DNase digestion. 1 µg of RNA was reverse transcribed using the Thermoscript RT PCR System (Invitrogen) and 50 mM oligo(dT)_20_ for priming. The newly synthesised cDNA was additionally treated with 2 units of RNase H at 37°C for 30 min.

Amplification of the target genes with standard RT-PCR was performed on cDNA amounts equivalent to either 100 ng or 10 ng of RNA input. A 50 µl reaction consisted of 1× PCR buffer, 1.5 mM MgCl_2_, 0.2 mM dNTP mix, 10 pmol of each of the corresponding primers (Table 1) and 1 U/reaction of Platinum® *Taq* DNA Polymerase (Invitrogen).

Quantitative real time PCR (qPCR) was carried out on a MyiQ Single-Color Real-Time PCR Detection System (BIORAD). The reaction volume was 25 µl containing cDNA diluted to the equivalent of 12.5 ng RNA, 10 pmol of each primer, and 12.5 µl of iQ SYBR Green Supermix (BIORAD). The thermal cycling conditions for all qPCR assays included an initial enzyme activation step at 95°C for 3 min, followed by 40 cycles of denaturation at 95°C for 10 s and annealing at 60°C for 30 s. The PCR for the housekeeping gene TATA-binding protein (TBP), which was found to have medium but constant/similar mRNA levels across the different melanoma cell lines, and the target genes were performed in parallel for each sample. All samples were run in triplicate and in 3 independent experiments. The primers used for the amplification of the target genes are listed in Table 1. Standard curves with four 10-fold serial dilutions (1×, 0.1×, 0.01× and 0.001×) were produced to ensure that the amplification efficiencies for all primer pairs used were 100%. The specificity of real-time PCR was verified by melting curve analysis. The mRNA levels of the target genes were normalized to the relative amounts of the housekeeping gene TBP. The 2^−ΔΔCt^ method was used to calculate the fold-relationships in gene expression between the tested melanoma lines [51].

### Construction and purification of GST-IL-24 fusion protein

For generation of GST-IL-24 (or synonymously GST-mda7) fusion proteins, full length IL-24 was amplified from RNA extracted with the RNeasy mini kit (Qiagen, Hilden, Germany) from HaCaT cells using RT-PCR primers listed in Table 1, which incorporated BamHI and XhoI restriction sites for cloning into pGEX-5X1 vector (Amersham, GE Healthcare). The fusion protein was intentionally similar to the one described by Sauane et al. (2004) and therefore the signal peptide was included although this should not exert any function when situated between the GST and the IL-24 moiety. GST fusion protein (or GST alone) was produced in BL21(DE3) bacteria. A pre-culture of 20 ml (LB containing 2% glucose and 50 ug/ml ampicillin) was grown at 37°C to an OD_600_ of 1.4 and then inoculated into 200 ml of LB (+2% glucose) medium. When the OD_600_ reached 0.4, temperature was dropped to 18°C. At an OD_600_ of 0.6, protein production was induced with 0.5 mM IPTG for 3 h at 18°C. Cells were harvested by 15 min centrifugation at 5000 rpm and the pellet was lysed in 40 ml lysis buffer (20 mM Tris pH7.5, 50 mM NaCl, 0.2% Igepal CA 630, 1 mg/ml lysozyme, 1 tablet complete protease inhibitor cocktail (Roche Biochemicals) in 50 ml lysis buffer) followed by 4 freeze (15 min dry ice)/thaw (10 min 37°C) cycles. 25 ug/ml DNase was added for 30 min at room temperature to the lysate, which was then clarified by 30 min centrifugation at 4°C. Subsequently, GST-containing proteins were affinity-purified by incubation with washed and equilibrated glutathione sepharose 4B slurry (Amersham Biosciences) (750 µl) overnight at 4°C. The sepharose was pelleted at 1000 rpm for 5 min (4°C) and washed 4×15 min with 7.5 ml PBS each. Fusion proteins were eluted with 500 µl elution buffer (10 mM reduced glutathione in 50 mM Tris-HCl, pH 8.0) for 90 min rotating at room temperature followed by a second and third elution for 45 min each. Quality and purity of fusion proteins was verified on Coomassie gels and by western blot. Protein concentrations were determined using a standard Bradford assay (Biorad).

### Construction of ER-targeted recombinant IL-24 proteins

For ease of subsequent cloning steps, we modified the pcDNA5/FRT/TO vector (Invitrogen) by introducing a new multiple cloning site (MCS) containing restriction sites for the following enzymes: BsiWI, AscI, SbfI, SgrAI, FseI, PacI, and SwaI. For this, oligos with HindIII and NotI overhangs were synthesised (Eurogentec, Belgium), annealed, and then ligated into HindIII- and NotI-opened pcDNA5/FRT/TO resulting in the modified vector pcDNAmod. Forward oligo: 5′AGCTTCGTACGAAGGCGCGCCTACCTGCAGGTTTCACCGGTGTAAGGCC GGCCTGATTAATTAAGTAATTTAAATGC, Reverse oligo: 5′GGCCGCATTT AAATTACTTAATTAATCAGGCCGGCCTTACACCGGTGAAACCTGCAGGTAGGCGCGCCTTCGTACGA. Using standard cloning procedures, full length IL-24 was inserted into pcDNAmod. For this, the target was PCR-amplified from pGEX-5X1/IL-24 (described above) using primers listed in Table 1, which had a SbfI and a SgrAI site incorporated resulting in pcDNA-IL-24. Accordingly, pcDNA-IL-20 was generated; the IL-20 sequence was amplified from a previously described vector: pCEP-FLAG-IL-20 [7]. To generate vectors that target IL-24 expression to the endoplasmic reticulum (ER), a KDEL retention signal sequence was inserted into the FseI/PacI-opened pcDNA-IL-24 via a synthesised and annealed oligo harbouring FseI and PacI overhangs. Forward oligo: 5′ CCGGTAGTAAAGATGAACTTTAATTAAT, Reverse oligo: 5′ TAATTAAAGTTCATCTTTACTACCGGCCGG. All generated recombinant plasmids were sequenced (GATC, Konstanz, Germany) to ensure correct orientations and sequences of inserts.

### Production and quantification of human IL-24 (Hek-IL-24)

Hek293 cells were transiently transfected with pCEP-FLAG vector, pCEP-FLAG-IL-20, or pCEP-FLAG-IL-24 vector as described before [7]. Three to four days after transfection, supernatants were harvested and supplemented with protease inhibitor cocktail (Roche Biochemicals, Switzerland). Supernatants containing the recombinant proteins IL-20 and IL-24 were affinity-purified and further concentrated using anti-FLAG M2 affinity gel (Sigma) according to instructions supplied with the product. Supernatants and cell pellets from transfected Hek cells were analysed by Western blot and ELISA. To determine the concentrations of IL-24, anti-IL-24 antibody (R&D Systems) was coated over night in PBS on Microlon ELISA plates (Greiner, Belgium). Plates were washed, blocked and incubated with serial dilutions of IL-24-containing supernatants for 2 h at room temperature (RT). After washing, biotinylated anti IL-24 antibody (R&D Systems) was added for 2 h at RT. Subsequent incubation with Streptavidin-HRP (20 min, RT) was followed by addition of Tetramethylbenzidine (TNB) substrate. Reactions were stopped with 2N H_2_SO_4_ and absorbances were measured at 450 nm. Concentrations of affinity-purified IL-24 ranged between 3–8 ng/µl.
